# The experience of point-of-care testing for influenza in Scotland in 2017/18 and 2018/19 – no gain without pain

**DOI:** 10.2807/1560-7917.ES.2020.25.44.1900419

**Published:** 2020-11-05

**Authors:** Elizabeth M Dickson, Diogo FP Marques, Sandra Currie, Annette Little, Kirsty Mangin, Michael Coyne, Arlene Reynolds, Jim McMenamin, David Yirrell

**Affiliations:** 1 European Public Health Microbiology Training Programme (EUPHEM), European Centre for Disease Prevention and Control (ECDC), Stockholm, Sweden; 2 Health Protection Scotland, Public Health Scotland, Glasgow, United Kingdom; 3 Department of Medical Microbiology, Ninewells Hospital, Dundee, United Kingdom

**Keywords:** Influenza, point-of-care testing, national surveillance, patient management

## Abstract

**Background:**

During the 2017/18 and 2018/19 influenza seasons, molecular amplification-based point-of-care tests (mPOCT) were introduced in Scotland to aid triaging respiratory patients for hospital admission, yet communication of results to national surveillance was unaccounted for.

**Aim:**

This retrospective study aims to describe steps taken to capture mPOCT data and assess impact on influenza surveillance.

**Methods:**

Questionnaires determined mPOCT usage in 2017/18 and 2018/19. Searches of the Electronic Communication of Surveillance in Scotland (ECOSS) database were performed and compared with information stored in laboratory information management systems. Effect of incomplete data on surveillance was determined by comparing routine against enhanced data and assessing changes in influenza activity levels determined by the moving epidemic method.

**Results:**

The number of areas employing mPOCT increased over the two seasons (6/14 in 2017/18 and 8/14 in 2018/19). Analysis of a small number of areas (n = 3) showed capture of positive mPOCT results in ECOSS improved between seasons and remained high (> 94%). However, capture of negative results was incomplete. Despite small discrepancies in weekly activity assessments, routine data were able to identify trend, start, peak and end of both influenza seasons.

**Conclusion:**

This study has shown an improvement in capture of data from influenza mPOCT and has highlighted issues that need to be addressed for results to be accurately captured in national surveillance. With the clear benefit to patient management we suggest careful consideration should be given to the connectivity aspects of the technology in order to ensure minimal impact on national surveillance.

## Introduction

Point-of-care tests (POCT) for influenza have been available since the late 1990s [1]. However, these were relatively insensitive tests relying on the detection of viral antigens. More recently, POCT using molecular nucleic acid amplification (mPOCT), which have increased sensitivity and are comparable to the gold standard laboratory PCR tests (hereafter named as laboratory-derived tests/results), have become available making them an attractive and acceptable option for frontline healthcare services. mPOCT have been implemented and validated within hospital settings [2-8], and community settings [9]. A study performed in 2019 reported that the use of mPOCT in an emergency department in London was associated with reduced nosocomial transmission of influenza [3]. Another study from the Netherlands documented a positive experience with mPOCT in one teaching hospital, reporting reduced turnaround times, improved patient flow and estimated savings of roughly EUR 400,000 [4].

Influenza surveillance is an important public health activity for ensuring that there are adequate health service resources available and appropriate interventions accessible, particularly for those who are at risk of complications of influenza [10,11]. In Scotland, influenza activity is monitored on a weekly basis during the winter period through a wide range of surveillance components. The national influenza surveillance is composed of laboratory results from diagnostic and reference laboratories. These are transferred electronically from individual laboratory information management systems (LIMS) to the Electronic Communication of Surveillance in Scotland (ECOSS) database, managed by Health Protection Scotland (HPS).

During the 2017/18 influenza season, there were moderate to high levels of influenza activity reported across Scotland, putting significant pressure on bed occupancy in an already stretched hospital system (data not shown). mPOCT were rapidly introduced in many of the 14 territorial health boards in Scotland as a means of triaging for hospital admission. The introduction of mPOCT was to supplement and not replace routine testing and therefore resulted in an increase in the total number of patients tested. This had a positive effect on local bed occupancy, treatment and infection control interventions [12]. However, due to the speed of introduction, provision had not necessarily been made to enable capture of the results to ECOSS, and the impact on national influenza surveillance in Scotland was potentially compromised. Prior to the start of the 2018/19 season, HPS attempted to assess and find ways to mitigate the loss of national data as experienced in the previous season. This retrospective study aims to describe the steps taken to capture mPOCT data, assess the impact on influenza surveillance and describe the potential public health challenges resulting from the mPOCT roll-out.

## Methods

### Setting and study population

Scotland is divided into fourteen territorial health boards (hereafter referred to as areas A-N), which collectively provide healthcare for ca 5.4 million inhabitants. Healthcare can be given at a number of institutions from general practices, community pharmacies, out-of-hours clinics and medical receiving hospitals. Any of these services could potentially offer mPOCT for influenza, but the services that used this technology in the 2017/18 and 2018/19 influenza seasons were acute hospital-based. The population studied was therefore the total number of patients that presented with influenza-like illness to an acute hospital-based service, were tested for influenza using mPOCT and had the results transferred to LIMS.

### mPOCT implementation questionnaire

At the end of the 2017/18 influenza season, a questionnaire was developed (Supplement 1) and sent to areas A-N to determine the scale of mPOCT implementation. This was followed by a teleconference with all respondents that reported the use of mPOCT for influenza. Information requested in the questionnaire included the test manufacturer, location of the testing unit, who carried out the tests, how quality assessment was performed, what testing protocols were followed and how the results were reported. This led to the development of a nationally agreed advisory statement in November 2018 on the preferred way to implement mPOCT [12]. In 2018/19, a similar questionnaire (Supplement 2) was distributed to areas A-N before the beginning of the influenza season, but more emphasis was placed on the transfer of mPOCT results to LIMS, whether manual entry of results was required and what codes were assigned to ensure identification of mPOCT.

### Analysis of data transfer from LIMS to ECOSS

Influenza laboratory results available in ECOSS were analysed and text fields searched to identify keywords or codes that would indicate that the influenza test was performed using mPOCT (as reported in the mPOCT implementation questionnaire). ECOSS records were then categorised as mPOCT positive or mPOCT negative results and aggregated to obtain weekly counts for each laboratory. Data were aggregated from week 40 2017 to week 20 2018 (season 2017/18) and from week 40 2018 to week 20 2019 (season 2018/19).

### Completeness of mPOCT data in ECOSS

Extracts of the equivalent LIMS mPOCT data for the above periods were requested from a small number of participating areas (n = 3) to assess the completeness of both positive and negative ECOSS mPOCT results. Completeness was calculated as:

 number of ECOSS mPOCT results (positive or negative) / number of LIMS mPOCT results (positive or negative).

### Impact of mPOCT on microbiological surveillance data

For each participating area, the impact of incomplete or lacking mPOCT data in the Scottish influenza surveillance was assessed by comparing ECOSS routine data (nationally agreed data electronically transferred from the local laboratory (LIMS), which may or may not include all mPOCT local laboratory-derived results) to ECOSS enhanced data (ECOSS data with mPOCT results identified through text searches were removed and replaced by the LIMS extracted mPOCT data to avoid double counting). In order to compare these data, we calculated two indicators: (i) proportion of positives (number of positive results divided by the number of tests performed) and (ii) rate of positives (number of positive results expressed per 100,000 population).

### Proportion of mPOCT vs laboratory-derived positive results and tests

We estimated mPOCT usage between the two seasons as a proportion of all positive test results i.e. laboratory-derived plus mPOCT (ECOSS enhanced data). For this calculation we divided the mPOCT figure provided by LIMS by the figure provided by ECOSS enhanced data.

### Statistical analysis

For each indicator value we calculated the Pearson correlation coefficient (r-value) and compared the respective influenza activity levels to investigate whether having complete mPOCT data would change our interpretation of influenza weekly activity. Weekly influenza activity level was defined using the moving epidemic method (MEM) [13]. MEM is a standardised method for reporting influenza activity adopted by the European Centre for Disease Prevention and Control that allows intra- and inter-country comparisons. We used MEM to calculate intensity thresholds and identify influenza activity levels based on the two indicators mentioned above (proportion of positives and rate of positives). The MEM thresholds were calculated using the ‘mem’ R package (R software version 3.5.1 (R Foundation, Vienna, Austria), and the package ‘mem’ version 2.14) using the predefined configuration, i.e. fixed criterium method and a slope parameter of 2.8. For each indicator, and based on historical data since the 2010/11 season, MEM defined the following weekly influenza activity levels [14]: baseline (data below epidemic threshold); low (data between epidemic and low thresholds); moderate (data between low and medium thresholds); high (data between medium and high thresholds); extraordinary (data above high threshold).

### Ethical statement

This study used only aggregate and non-identifiable data, therefore no ethical approval was necessary.

## Results

### mPOCT implementation questionnaire

During the 2017/18 influenza season, six of 14 areas reported use of influenza mPOCT compared with eight of 14 in the 2018/19 season (Supplement 3). The majority of mPOCT were used at acute hospital admissions or emergency departments during the 2017/18 season, with more specialised departments (e.g. oncology and paediatric ICU) using mPOCT during the 2018/19 season. With the exception of outlying hospitals where testing was performed by laboratory staff, the majority of mPOCT were performed by ward staff.

According to additional comments received in the questionnaires, training in the first instance was usually performed by the mPOCT manufacturers, with some departments supplementing this with training by laboratory staff. Quality assessment was minimal due to time and cost restraints, which led to shorter verification processes and general acceptance of the manufacturer’s sensitivity and specificity claims. All areas agreed a local protocol with clinicians as to who should be tested and under what circumstances. It was noted that during the 2017/18 season this was not always adhered to and an increase in number of tests was reported due to testing of asymptomatic individuals, contacts or members of staff. In all cases, patient management decisions were based entirely on the result of the mPOCT, including use of personal protective equipment (PPE), antiviral treatment, admission and transfer.

In the 2017/18 influenza season, none of the areas had direct transfer of test results from the mPOCT machine to their LIMS, thus data transfer was performed manually (frequency variable). In the 2018/19 influenza season, despite differences between areas, manual entry of mPOCT results was required at some stage of the data transfer process. Of note, two areas used a central computational system (middleware), which received data from multiple mPOCT machines before transferring to LIMS. However, this link did not work and manual data extraction from the middleware was required. In addition, information received from the 2018/19 questionnaire showed there was no consistent use of identifiable mPOCT codes across areas.

### Analysis of data transfer from LIMS to national database

The analysis of ECOSS data only identified a small number of areas that had records categorised as mPOCT based on text searches (two of six in 2017/18: areas D and F; and three of eight in 2018/19: areas D, F and M). Due to this, only these areas were further analysed and investigated for completeness. The weekly aggregated counts for the 2017/18 and 2018/19 seasons were then requested from these areas. Among areas D, F and M, only area F had negative results available through ECOSS, but this was due to the laboratory in this area having pre-existing data transfer in place for all influenza test results.

### Completeness of mPOCT data in ECOSS

The results presented in Table 1 show the proportion of mPOCT positive results captured by ECOSS i.e. completeness increased between the 2017/18 and 2018/19 seasons for areas D and F by 5.2% and 32.4%, respectively. In the 2018/19 season, a very high proportion of mPOCT positive results was captured by ECOSS (93.8%, 96.7% and > 100% for areas D, F and M, respectively). The proportion of mPOCT negative results captured by ECOSS for area F was very low in both seasons (7.3% and 4% in 2017/18 and 2018/19, respectively). It was not possible to calculate the completeness of mPOCT negative results in ECOSS for areas D and M as influenza negative results from laboratories in these areas were not routinely captured by ECOSS. Discrepancies in mPOCT results between ECOSS and LIMS, as seen in area M, were determined to be a result of differences in coding, subsequent incorrect data entry into text fields, and the code not being recognised by the software during data extraction.

**Table 1:** Completeness of positive and negative influenza mPOCT results transferred from LIMS to ECOSS, Scotland, influenza seasons 2017/18 and 2018/19

mPOCT result	Area	Season 2017/18^a^			Season 2018/19^a^			
		ECOSS mPOCT	LIMS mPOCT	Completeness (%)	ECOSS mPOCT	LIMS mPOCT	Completeness (%)^b^	DIFF
Positive	D	164	185	88.6	227	242	93.8	5.2
	F	539	838	64.3	559	578	96.7	32.4
	M	NA	NA	NA	202	199	> 100	–
Negative^c^	D	NA	NA	NA	NA	NA	NA	–
	F	86	1,172	7.3	53	1,333	4.0	−3.4
	M	NA	NA	NA	NA	NA	NA	–

DIFF: difference; ECOSS: Electronic Communication of Surveillance in Scotland database; LIMS: laboratory information management system; mPOCT: molecular amplification-based point-of-care tests; NA: not available.

^a^ Results were obtained from tests carried out from week 40 2017 to week 20 2018 (influenza season 2017/18) and from week 40 2018 to week 20 2019 (influenza season 2018/19).

^b^ Completeness of data transferred from individual LIMS to the ECOSS database. Completeness calculated as number of ECOSS mPOCT results (positive or negative) / number of LIMS mPOCT results (positive or negative).

^c^ Negative results were generally not transferred to ECOSS. They were available for area F due to the laboratory in this area having pre-existing data transfer in place for all influenza test results.

### Impact of mPOCT on influenza surveillance data

**Figure 1:**
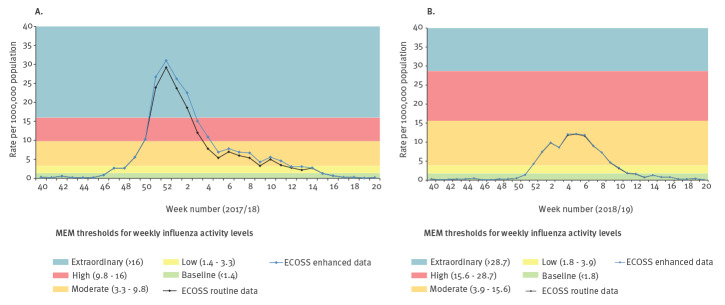
Comparison of rate of positive influenza tests per 100,000 population between the ECOSS routine data and ECOSS enhanced data collection methods for area F during (A) influenza season 2017/18 and (B) influenza season 2018/19, Scotland

ECOSS: Electronic Communication of Surveillance in Scotland database; MEM: moving epidemic method.

**Figure 2:**
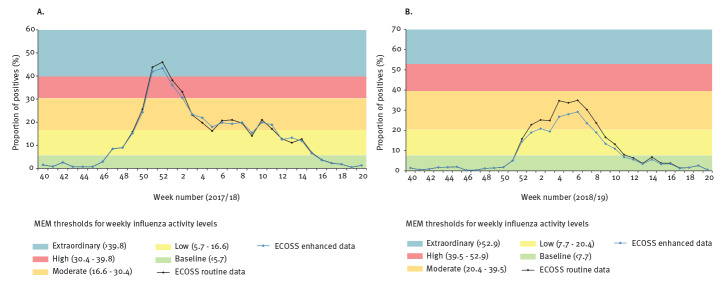
Comparison of proportion of positive^a^ influenza tests between the ECOSS routine data and ECOSS enhanced data collection methods for area F during (A) influenza season 2017/18 and (B) influenza season 2018/19, Scotland

ECOSS: Electronic Communication of Surveillance in Scotland database; MEM: moving epidemic method.

^a^ Proportion of positives calculated as number of positive tests / number of total tests.

Data obtained from area F were used to assess the impact of mPOCT in influenza surveillance as it enabled the analysis of both positivity rate and proportion of positives (required negative results). In terms of rate of positives, the weekly influenza activity level based on ECOSS enhanced data was similar to that of ECOSS routine data (Figure 1). Both seasons’ data showed a very high correlation coefficient (0.996 and 0.999 in season 2017/18 and 2018/19, respectively). This is in line with the high proportion of positives being captured by ECOSS in both seasons (Table 1). The low proportion of mPOCT negatives being captured by ECOSS meant an overestimation of the proportion of positives calculated during the two seasons (Figure 2), i.e. the ECOSS routine data showed an artificially higher proportion of positives than the ECOSS enhanced data. However, the overall trends remained similar and influenza activity level interpretation was similar for both ECOSS datasets for most weeks. There was one week in the 2017/18 season (Figure 2A) where level interpretation would have been moderate instead of low if we were using ECOSS enhanced data (week 5 2018). In the 2018/19 season, if we were using the ECOSS enhanced data there would have been 3 weeks where activity level would have been low instead of moderate (weeks 1,3, and 8 in 2019) and one week where activity level would have been baseline instead of low (week 11 2019) (Figure 2B). Despite these sporadic discrepancies, the data available in ECOSS for area F allowed the identification of the start, peak and end of both influenza seasons. The intensity of the peak and the timing of the start and end of the seasons were the same regardless of using ECOSS routine or ECOSS enhanced data.

Results were obtained from tests carried out from week 40 2017 to week 20 2018 (influenza season 2017/18) and from week 40 2018 to week 20 2019 (influenza season 2018/19).

Pearson correlation coefficient between ECOSS routine and ECOSS enhanced data was (A) (r = 0.996) and (B) (r = 0.999).

Results were obtained from tests carried out from week 40 2017 to week 20 2018 (influenza season 2017/18) and from week 40 2018 to week 20 2019 (influenza season 2018/19).

Pearson correlation coefficient between ECOSS routine and ECOSS enhanced data was (A) (r = 0.996) and (B) (r = 0.998).

### Proportion of mPOCT vs laboratory-derived positive results and tests

There was an increase in the proportion of mPOCT among all tests performed (laboratory-derived and mPOCT together) in area F from 15.5% to 17.7% in the 2017/18 and 2018/19 season, respectively (Table 2). In addition, the proportion of mPOCT positive results among all positive results increased from 34% (2017/18 season) to 48% (2018/19 season). An increase in the proportion of mPOCT positive results among all positive results was also seen in area D (19% to 42% in the 2017/18 and 2018/19 season, respectively), and in the 2018/19 season the proportion of mPOCT positive results for area M was 45.2% (Table 2).

**Table 2:** Proportion of influenza mPOCT among all influenza tests^a^ performed in area F by influenza season, and proportion of positive influenza mPOCT among all positive influenza tests^a^ for areas F, M and D by season, Scotland, influenza seasons 2017/18 and 2018/19

Tests	Area	Season 2017/18					Season 2018/19				
		ECOSS mPOCT tests	LIMS mPOCT tests	ECOSS routine tests^a^	ECOSS enhanced tests^b^	Proportion of mPOCT tests (%)^c^	ECOSS mPOCT tests	LIMS mPOCT tests	ECOSS routine tests^a^	ECOSS enhanced tests^b^	Proportion of mPOCT tests (%)^c^
All tests	F	625	2,010	11,571	12,956	15.5	612	1,911	9,511	10,810	17.7
Positive tests	F	539	838	2,166	2,465	34.0	559	578	1,191	1,210	47.8
	M	NA	NA	NA	NA	NA	202	199	443	440	45.2
	D	164	185	939	960	19.3	227	242	567	582	41.6

ECOSS: Electronic Communication of Surveillance in Scotland database; LIMS: laboratory information management system; mPOCT: molecular amplification-based point-of-care tests; NA: not available.

^a^ Laboratory-derived + mPOCT.

^b^ (ECOSS routine − ECOSS mPOCT) + LIMS mPOCT.

^c^ LIMS mPOCT tests divided by ECOSS enhanced tests.

Results were obtained from tests carried out from week 40 2017 to week 20 2018 (influenza season 2017/18) and from week 40 2018 to week 20 2019 (influenza season 2018/19).

Proportion of mPOCT tests are a potential proportion due to not all LIMS mPOCT results being reported to ECOSS.

## Discussion

The 2017/18 influenza season was dominated by influenza A(H3N2) which is more likely to affect the elderly population [15], but with a noticeable tail of Influenza B cases which affects both the young and the elderly [16]. mPOCTs were rapidly introduced by health boards across Scotland and this study reports the consequential difficulties of this technology for the national microbiological surveillance of influenza.

Although different mPOCT systems were used in the different areas, the principal technology is the same and therefore does not affect the results. Most hospitals wanted to link the mPOCT machines directly to their LIMS. However this is technically difficult, requires time and local IT support, and is often expensive as LIMS providers charge for changes to their systems. Inevitably, in almost all cases an mPOCT machine to LIMS link was not established. Following the 2017/18 season, areas were encouraged to include a code in their mPOCT results and report this to HPS in order to enable differentiation between mPOCT generated results from laboratory results. However, this failed to be achieved in the 2018/19 season, possibly be due to the speed at which mPOCT were implemented, and compatibility issues between the different systems used.

Data from a small number of areas suggest that in most instances the positive cases of influenza are being captured by the national database (ECOSS). This is in contrast with the negative results where there is a sizeable gap between local and national figures. Incomplete mPOCT negative results data in the national surveillance system will overestimate the proportion of positives and potentially overestimate the weekly influenza activity levels. Despite this, data for area F showed that the differences in weekly influenza activity level were minimal and the existing microbiological surveillance was able to identify the trend, start, peak and end of the influenza epidemics in the 2017/18 and 2018/19 seasons. mPOCT accounted for ca 18% of all tests undertaken in area F, and up to 48% of all influenza positive results. The increased use of mPOCT and increased number of positive results reinforces the need for accurate data capture at national level. Work is ongoing and HPS along with the Scottish Microbiology and Virology Network (SMVN) are working with laboratories to standardise and improve data collection. However, the decision on which mPCOT machine to choose, and how to transfer the data is both laboratory and resource dependent.

It is important to note that, in addition to the microbiological surveillance, the national influenza surveillance is composed of other components such as calls concerning respiratory problems to the National Health Service (NHS) 24 helpline, GP consultation rates for influenza-like illness, outbreaks, severe acute respiratory illness and mortality surveillance. These are essential not only to capture the influenza burden in different parts of the population/healthcare but also to complement each other when there are changes in the surveillance system, such as the introduction of mPOCT.

The data presented here are the first that we are aware of that attempt to quantify the impact that mPOCT for influenza has had on the information being received by public health authorities. We have shown the importance of recognising what mPOCT results should be recorded. All users need to be aware of the impact that each of the variables will have on the estimation of proportion of positives, and how the data are used to assess influenza activity both at local and national level. The main challenge is capturing the mPOCT negative results within ECOSS in order to have an accurate denominator and to avoid overestimating the proportion of positives indicator. With this evidence now available, it is hoped that many of these issues can be addressed for future influenza seasons. While reporting from a Scottish perspective we anticipate that our observations are likely to reflect common issues found in other European countries in which the introduction of mPOCT for influenza pose a challenge for data recording, and as a consequence, the accuracy and completeness of surveillance information.

### Limitations

There are a number of caveats to our data and it is important to highlight them as a way of suggesting areas for consideration and improvement when planning implementation of mPOCT. This study covered only a sample of areas in Scotland therefore the total impact of incomplete or lack of mPOCT data in the national surveillance system (ECOSS) is still unknown. The impact is likely to be larger if the use of mPOCT increases dramatically and accounts for the majority of influenza tests. It is also important to stress that frontline users, e.g. nursing staff, may not always recognise the critical nature of recording and reporting every mPOCT result. Further work is required to quantify this and to identify laboratory-specific challenges that will need to be addressed. While ECOSS enhanced data were calculated to avoid double counting any mPOCT results, there may still be some instances of duplication in which identifiers were close, but not exactly matching. It was noted that during the 2017/18 season, adherence to local protocols was not always evident and some testing was performed on asymptomatic individuals, contacts or members of staff, although this number was minimal. There were a number of areas that were subject to manual data entry, which carries a risk of transcription errors. In order to minimise this risk, we recommend that all steps are automated and linked to the LIMS and ECOSS. The method of identifying an mPOCT via text searches in ECOSS is suboptimal and there is the potential for misclassification. The use of MEM applied to microbiological surveillance data depends on historical data as described elsewhere [17]. The lack of reliable and complete microbiological data (including mPOCT) can therefore limit the potential application of this methodology to assess influenza activity at local level where data might be limited.

In order to address some of the issues that have been discussed, a separate programme to improve all microbiology data received at HPS is currently underway. The ECOSS Data Roll Out Improvement Project (EDRIP) will review all data received from all NHS clinical microbiology laboratories in Scotland, including mPOCT data, over a two-year period. It is hoped that any issues identified will be addressed quickly to result in a continuous improvement to the quality of all data held within ECOSS. This will ensure that the impact of the influenza mPOCT programme in Scotland can be reliably assessed, and effectiveness of any interventions monitored.

### Conclusion

Through close liaison with the Scottish territorial health boards and respective laboratories, we have shown there was an improvement in mPOCT data collection between the 2017/18 and 2018/19 influenza seasons. Further work is needed to ensure accurate numbers of positive and negative mPOCT results are collected in ECOSS, including set up of direct LIMS connectivity, education of frontline users on the impact of missing results, and continued development and audit of local protocols. Due to the benefits for patient management, the use of mPOCT for influenza is likely to continue and implementation of these systems should be carefully managed in order to reduce the impact on national microbiological surveillance.

empirical study and modelling is required to optimise their use for public health benefit.

## Supplementary Data

566000 Supplement1

## Supplementary Data

301000 Supplement2

## Supplementary Data

529000 Supplement3
