# Supplementary material for: The experience of point-of-care testing for influenza in Scotland in 2017/18 and 2018/19 – no gain without pain
Source: Euro Surveill. 2020 Nov 5;25(44):1900419. doi: 10.2807/1560-7917.ES.2020.25.44.1900419 (PMC7645975; doi:10.2807/1560-7917.ES.2020.25.44.1900419)
Supplement: Supplement1 [file 1900419_DICKSON_Supplement1.pdf]

Supplement 1: Respiratory questionnaire 2017

This supplementary material is hosted by *Eurosurveillance* as supporting information alongside the article "The experience of point-of-care testing for influenza in Scotland in 2017/18 and 2018/19 – no gain without pain" on behalf of the authors who remain responsible for the accuracy and appropriateness of the content. The same standards for ethics, copyright, attributions and permissions as for the article apply. *Eurosurveillance* is not responsible for the maintenance of any links or email addresses provided therein.

Respiratory Point of Care (PoC) testing, winter 2017/2018

Every winter the arrival of Flu presents difficulties both to the NHS in general and laboratories in particular. The timing, duration and severity of each year is unpredictable and the pressure on clinical services dictates what pressure subsequently is put on diagnostic services.

This year, predicted to be a "big" flu year did not disappoint, arriving over the Christmas period. As pressures mounted rapid flu testing using point of care tests (PoC) was increasingly deployed in some hospitals.

In an attempt to inform subsequent years on the benefits of PoC tests HPS are gathering data on their use in Scotland.

If your lab was involved in flu PoC testing this winter, please could you to take a few minutes to complete the questionnaire below and return it by **Friday 13th April** to XXXXXXXXXXXXXXXXX

Many thanks for your time.

<u>Type of PoC Testing and Validation</u>

- What POC test(s) was used e.g. manufacturer and pathogen range?
- What funding model was used? eg reagent rental
- Did the laboratory develop a business case before PoC test installation?

(if yes, would you be happy to share your business case to inform a national approach?)

- Was your PoC test validated locally before installation?

(If yes would you be happy to share results?)

- What EQA and IQC testing is carried out?

- Is your PoC testing on your UKAS scope of practice?

Supplement 1: Respiratory questionnaire 2017

Location of PoC Testing and Training

- Where were PoC tests located? (List all areas e.g. paediatrics, acute admissions, other hospitals etc.)
- Who carried out the PoC testing?
- Was training provided to these staff? If yes,
    - When was training provided e.g. on an ongoing regular/refresher basis or only when PoC testing set up in the test location?
    - What training was provided and by whom?
    - Was user competency assessed?

PoC Testing and local protocols

- When was PoC testing started and stopped e.g. October to March or decided by local/national prevalence?
- What criteria are in place for deciding:
    - Location(s) for PoC testing?
    - Who to test?
    - What to test for?
    - When to test?
- Do you have an agreed, written, protocol covering the points above?
- Does this protocol include care pathways for patient's positive/negative for a respiratory virus on PoC Testing?
- What, if any, patient treatment/management decisions were made solely on the result of the PoC test?
- Was the PoC test directly linked to your laboratory LIMS?

  (if No, did you enter your PoC results onto your LIMS?)
- Were specimens tested by PoC re-tested again in the lab e.g. does that include only the positives, only the negatives, all specimens, or never?
- Were PoC positives reported to HPS or captured directly by ECOSS?
- If PoC tests are not shared with HPS currently do you have CHI listed data on the use of these PoC tests with date of test and result that can be shared with HPS electronically (e.g. excel, csv etc)?

Supplement 1: Respiratory questionnaire 2017

- For next season assuming that the results of the PoC tests are not included in ECOSS would you be able to submit weekly CHI listed data with results to HPS?
- In your experience to date how useful have the PoC tests been and what are their limitations?
