# Supplementary material for: The experience of point-of-care testing for influenza in Scotland in 2017/18 and 2018/19 – no gain without pain
Source: Euro Surveill. 2020 Nov 5;25(44):1900419. doi: 10.2807/1560-7917.ES.2020.25.44.1900419 (PMC7645975; doi:10.2807/1560-7917.ES.2020.25.44.1900419)
Supplement: Supplement2 [file 1900419_DICKSON_Supplement2.pdf]

Supplement 2: Respiratory questionnaire 2018

This supplementary material is hosted by *Eurosurveillance* as supporting information alongside the article "The experience of point-of-care testing for influenza in Scotland in 2017/18 and 2018/19 – no gain without pain" on behalf of the authors who remain responsible for the accuracy and appropriateness of the content. The same standards for ethics, copyright, attributions and permissions as for the article apply. *Eurosurveillance* is not responsible for the maintenance of any links or email addresses provided therein.

HPS Respiratory questionnaire 2018:

1) Is your health board using a flu POCT testing system?

2) If yes please detail

POCT System	Location	Connected to LIMS (Y/N)	Manual entry of POCT results (Y/N)	Code *	Comments

*Please enter code used to identify POCT results for identification/audit purposes

3) Is your POCT part of your UKAS scope of practice?

4) What is your policy for retesting patients screened by POCT?

5) Do you have written clinical and testing protocols that describes how the Flu POCT should be used, if so, can they be shared with HPS please?
