# Supplementary material for: The experience of point-of-care testing for influenza in Scotland in 2017/18 and 2018/19 – no gain without pain
Source: Euro Surveill. 2020 Nov 5;25(44):1900419. doi: 10.2807/1560-7917.ES.2020.25.44.1900419 (PMC7645975; doi:10.2807/1560-7917.ES.2020.25.44.1900419)
Supplement: Supplement3 [file 1900419_DICKSON_Supplement3.pdf]

Supplement 3: Data on Influenza POCT usage collected from 14 areas representing the 14 territorial health boards in Scotland

This supplementary material is hosted by *Eurosurveillance* as supporting information alongside the article "The experience of point-of-care testing for influenza in Scotland in 2017/18 and 2018/19 – no gain without pain" on behalf of the authors who remain responsible for the accuracy and appropriateness of the content. The same standards for ethics, copyright, attributions and permissions as for the article apply. *Eurosurveillance* is not responsible for the maintenance of any links or email addresses provided therein.

	Is lab using a POCT system?		What system?		Location		Who performs test?		LIMS connection in place?		Is this part of your labs UKAS* scope of practice?		Do you have written testing protocols?	
	2017/18	2018/19	2017/18	2018/19	2017/18	2018/19	2017/18	2018/19	2017/18	2018/19	2017/18	2018/19	2017/18	2018/19
**Area A**	No	No												
**Area B**	No	No												
**Area C**	No	Yes		Cepheid GeneXpert[1]		Microbiology				No		Intended		Local protocol in place
**Area D**	Yes	Yes	Cepheid GeneXpert[1]	Roche Liat[1]	ED	ED, AMU	Nursing staff	Nursing staff	No	Yes	No	No	Yes	Yes
**Area E**	Yes (Trial basis)	Yes	Cepheid GeneXpert[1]	Cepheid GeneXpert[1]	Clinical assessment unit	ED	Nursing staff	Nursing staff	No	No	No	No response	Yes	Yes
**Area F**	Yes	Yes	Cepheid GeneXpert[1]	Roche Liat[1]	Oncology, 3 EDs, 1 ICU, 2 AU	Oncology, 2 EDs, 3 ICU (incl paed), 1 IDU and 3 AU	Trained staff	Trained staff	No	No	No	Not yet	Local agreement	Draft guidelines
**Area H**	Yes	Yes	GenMark ePlex[2]	Genmark ePlex[2]	4 AU	3 AU	Nursing staff or lab staff		No	No	Awaiting extension to scope	UKAS accredited for lab generated results	Yes	Yes but due to be reviewed

[Type text]

Supplement 3: Data on Influenza POCT usage collected from 14 areas representing the 14 territorial health boards in Scotland

**Area G**	Yes (Trial basis)	No	GenMark ePlex[2]											
**Area H**	Yes	Yes	GenMark ePlex[2]	Genmark ePlex[2]	4 AU	3 AU	Nursing staff or lab staff		No	No	Awaiting extension to scope	UKAS accredited for lab generated results	Yes	Yes but due to be reviewed
**Area I**	No	Yes		Roche Liat[1]		ARU				Yes		No response		No response
**Area J**	Yes	Yes	Cepheid GeneXpert[1] & GenMark ePlex [2]	Cepheid GeneXpert[1] & GenMark ePlex[2]	Various including 2 AU, 2 EDs	Cepheid: 1 ED, 1 ARU. GenMark: oncology, paed AU	Trained staff		No	No	No	Not yet	Yes	Yes
**Area K**	No	No												
**Area L**	No	No												
**Area M**	No	Yes		Roche Liat[1]		1 AMU, 1 AU		Nursing staff		Yes		No		Yes
**Area N**	No	TBD†												

[1]narrow range of pathogens tested; [2]panel of respiratory pathogens tested; *United Kingdom Accreditation Service; †TBD - to be determined

ED - emergency department; AMU - acute medical unit; ICU - intensive care unit; ARU - acute receiving unit; AU - admissions unit; IDU - infectious diseases unit; paed - paediatrics
